# Epacadostat and Olaparib Synergistically Inhibit the Growth of BRCA-Proficient Triple-Negative Breast Cancer by Suppressing the Expression of BRCA1 and RAD51

**DOI:** 10.3390/molecules31061039

**Published:** 2026-03-20

**Authors:** Lei Huang, Ye Yang, Dongxia Duan, Li Dai, Bingxin Zhai, Bingjun Qian

**Affiliations:** 1 Department of Pharmacology and Medicinal Chemistry, Jiangsu Medical College, Yancheng 224005, China; 2 Department of Preventive Medicine, Jiangsu Medical College, Yancheng 224005, China; 3 Department of Medicinal Chemistry, China Pharmaceutical University, Nanjing 210009, China

**Keywords:** IDO1, PARP1/2, HR, NAD^+^, ROS, BRCA-proficient, TNBC

## Abstract

Triple-negative breast cancer (TNBC) cells with intact homologous recombination (HR) repair mechanism can survive treatment with Olaparib, which further limits the clinical application of PARP1/2 inhibitors. Previous studies have demonstrated that inhibition of indoleamine 2,3-dioxygenase (IDO) can enhance the sensitivity of human tumor cells to PARP1/2 inhibitors. However, the mechanisms underlying their synergistic effects in the treatment of TNBC remain unclear. Herein, we demonstrate that the combination of Olaparib and Epacadostat significantly reduces the proliferation of BRCA-proficient MDA-MB-231 and MDA-MB-468 cells compared to either monotherapy. Mechanistically, Epacadostat reduces intracellular kynurenine and NAD^+^ levels, thereby sensitizing TNBCs to PARP1/2 inhibition and significantly amplifying Olaparib-induced DNA damage. Furthermore, Epacadostat and Olaparib synergistically increase cellular reactive oxygen species (ROS), leading to DNA oxidative damage and apoptosis. In vivo, Epacadostat and Olaparib significantly suppressed MDA-MB-468 tumor growth compared to the monotherapy groups, while promoting an increase in phosphorylated H2AX. Notably, the dual inhibition of IDO1 and PARP1/2 specifically reduced the expression of HR core genes and proteins, such as BRCA1 and RAD51, which may contribute to impaired DNA-damage repair and increased sensitivity to Olaparib. In summary, targeting both IDO1 and PARP1/2 represents a promising combination therapy for BRCA-proficient TNBC.

## 1. Introduction

Triple-negative breast cancer (TNBC) accounts for 10–15% of all breast cancer cases and poses a significant therapeutic challenge, with a 5-year survival rate below 20% [1]. Due to the absence of estrogen receptors, progesterone receptors, and human epidermal growth factor receptor 2 (HER2) expression, traditional endocrine therapies and HER2-targeted agents are ineffective against TNBC [2,3]. Currently, chemotherapy remains the standard treatment regimen for TNBC patients, but its efficacy is significantly limited by toxic side effects and drug resistance [4]. Consequently, the development of novel therapeutic strategies for TNBC is imperative.

Recent advancements in molecularly targeted therapies and immunotherapies have provided diverse treatment options for TNBC patients. Notably, the success of Poly (ADP-ribose) polymerase 1/2 (PARP1/2) inhibitors in treating TNBC with BRCA1/2 mutations, achieved by targeting the DNA damage response (DDR), has raised promising prospects for clinical treatment [5]. Targeting the DDR induces synthetic lethality in tumor cells without affecting normal tissue, indicating a promising strategy for improving cancer treatment [6]. DNA damage typically includes DNA single-strand breaks (SSBs) and DNA double-strand breaks (DSBs), in addition to base changes [7]. PARP1/2 primarily participates in the repair of single-strand breaks through base excision repair (BER) [8]. Conversely, homologous recombination repair (HRR) has been identified as the most effective method for the precise repair of DNA DSBs. During the HRR process, the proteins BRCA1/2 and RAD51 are recruited to the damaged DNA for precise repair [9]. In circumstances where the HRR pathway is impaired, the inhibition of PARP1/2 enzyme activity gives rise to the accumulation of unrepaired DNA DSBs in tumor cells. This, in turn, rapidly induces tumor cell death. The principle of synthetic lethality has been utilized in the development of several PARP1/2 inhibitors, which have been approved for the treatment of patients with BRCA1/2 mutations [10]. However, only a small subset of TNBC patients exhibits HRR deficiency, which limits the broader clinical application of PARP1/2 inhibitors [11]. Inspired by the concept of synthetic lethality, targeting novel target or signaling pathways to induce HRR deficiency might broaden the therapeutic benefits of PARP1/2 inhibitors for a larger population of TNBC patients.

Currently, the exploration of novel targets to induce HRR deficiencies is a primary strategy to expand the indications of PARP1/2 inhibitors. Emerging evidence indicates that indoleamine 2,3-dioxygenase (IDO) plays a role in DNA damage repair, reactive oxygen species (ROS), the cell cycle, and immunity [12,13,14]. IDO1, a member of the oxygenase superfamily, has been observed to be overexpressed in diverse malignancies, including TNBC. Recent studies have shown that the knockdown of IDO1 via shRNA or the use of IDO1 inhibitors can significantly increase intracellular ROS levels in tumor cells, which directly downregulates the expression of HRR-related proteins, such as RAD51, BRCA1 and BRCA2 [15,16]. Saman Maleki Vareki et al. (2014) reported that overexpression of IDO1 in cancer cells stimulated by IFN-γ can reduce the therapeutic efficacy of PARP1/2 inhibitors, while knockout of the IDO1 gene enhances the effectiveness of PARP1/2 inhibition [12]. Furthermore, IDO1 catalyzes the conversion of L-tryptophan to kynurenine, subsequently promoting the synthesis of the metabolite nicotinamide adenine dinucleotide (NAD^+^), which plays a critical role in the PARP-mediated DNA repair pathway [17,18]. Li et al. (2017) demonstrated that a reduction in NAD^+^ facilitates the binding of DBC1 to PARP1, leading to the accumulation of DNA damage, and that this process can be rapidly reversed by restoring the abundance of NAD^+^ [19]. Accordingly, we speculated that inhibiting IDO1 function may enhance the sensitivity of BRCA-proficient TNBC cell lines to PARP1/2 inhibitors.

In this study, we selected the IDO1 inhibitor Epacadostat and the PARP1/2 inhibitor Olaparib to investigate their synergistic effects on BRCA-proficient TNBC cell lines and the underlying mechanisms both in vitro and in vivo.

## 2. Results

### 2.1. Epacadostat and Olaparib Synergistically Inhibit the Growth of BRCA-Proficient TNBC Cells In Vitro

**Figure 1:**
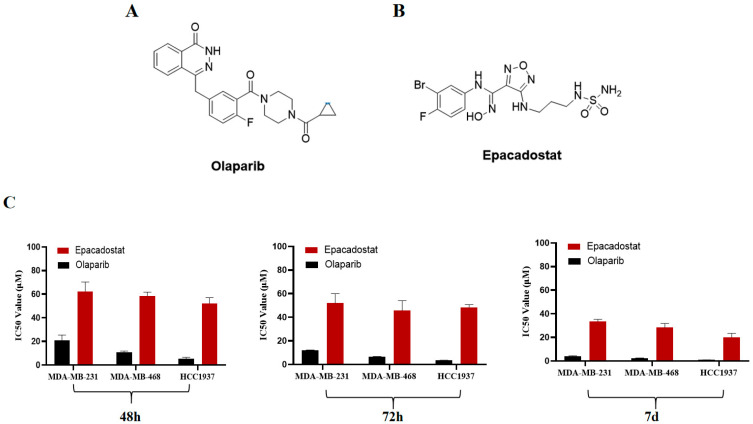

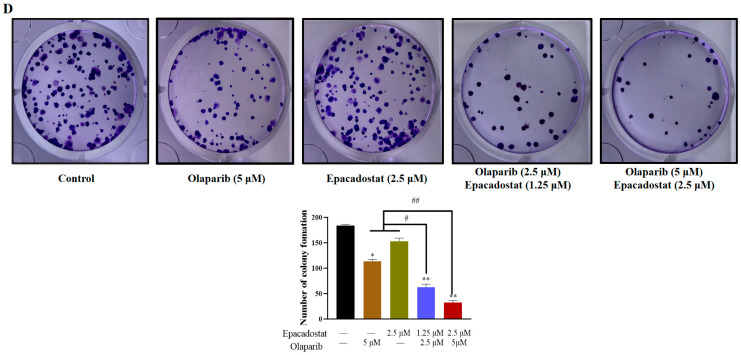
Synergistic effects of Epacadostat or/and Olaparib on the growth of TNBC cells. (**A**) The structure of the PARP1/2 inhibitor Olaparib. (**B**) The structure of the IDO1 inhibitor Epacadostat. (**C**) IC_50_ values for the effects of Epacadostat or Olaparib on the viability of MDA-MB-231, MDA-MB-468 and HCC1937 cells measured by CCK-8 assay at different time points. (**D**) The effects of Epacadostat and/or Olaparib on the proliferation of MDA-MB-468 cells assessed using a colony-forming assay. “-” means the compound was not added. Statistical significance of differences was determined by one-way ANOVA. * *p* < 0.05 and ** *p* < 0.01 compared with control group, ^#^ *p* < 0.01 and ^##^ *p* < 0.01 compared with the combination group of Epacadostat and Olaparib.

To evaluate the potential of IDO1 as a valuable target, an analysis was conducted of the differential expression of IDO1, PARP1, and PARP2 between cancer tissues and adjacent normal tissues in breast cancer. The analysis was based on data from the Cancer Genome Atlas (TCGA) project. The results indicated that IDO1 and PARP1 are overexpressed in TNBC tissues (Figure S1A). Furthermore, the increased expression of IDO1 has been shown to be significantly correlated with worse overall survival (OS) and disease-free survival (DFS) in patients with TNBC (Figure S1B–E). To assess further whether the inhibition of IDO1 could result in the sensitization of BRCA-proficient TNBC cell lines to the PARP1/2 inhibitor, the IDO1 inhibitor Epacadostat and PARP1/2 inhibitor Olaparib were selected for cell counting kit-8 (CCK-8) analysis (Figure 1A,B). As shown in Figure 1, the inhibitory effects of Epacadostat or Olaparib on the growth of MDA-MB-231, MDA-MB-468 and HCC1937 cells exhibited a time-dependent increase. Following a 7-day treatment period, Olaparib inhibited the growth of all three TNBC cell lines; however, the inhibition was significantly stronger in BRCA1-mutated HCC1937 cells than in BRCA-proficient MDA-MB-231 and MDA-MB-468 cells (Figure 1C and Figure S2A–C). Subsequently, the synergistic effects of Epacadostat, Olaparib, and their fixed-dose combination on the proliferation of two additional BRCA-proficient TNBC cell lines were investigated. The results indicated that the combination of Epacadostat and Olaparib exhibited a substantial growth inhibition effect on the MDA-MB-231 and MDA-MB-468 cell lines compared with either monotherapy. Following comprehensive analysis of the combination index (CI), it was determined that the synergistic effect (CI < 1.0) of Epacadostat and Olaparib on the growth of BRCA-proficient TNBC cells was observed to be dependent upon the dose ratio of the two drugs ranging from 1:1 to 1:2. (Table 1 and Table 2). More importantly, a 1:2 dose ratio of Epacadostat to Olaparib was found to be most effective in exerting their synergistic effects in MDA-MB-468 cells. Therefore, this ratio was selected for further investigation. Furthermore, treatment with Epacadostat or Olaparib did not significantly affect the viability of the normal human mammary epithelial cell line MCF10A (IC_50_ > 50 μM). No synergistic effect was observed (CI > 1.0) in the combination therapy, suggesting a selective inhibition of tumor cells (Figure S3). Finally, a colony formation assay was conducted to validate the synergistic effects of Epacadostat and Olaparib at a 1:2 ratio on the proliferation of MDA-MB-468 cells. The results demonstrated that Epacadostat and Olaparib exhibited a substantial inhibition of colony formation in a concentration-dependent manner (Figure 1D). Notably, a similar synergistic inhibitory effect of Epacadostat and Olaparib was also observed in the BRCA-proficient pancreatic cancer cell line SW1990, suggesting that this combination strategy may be applicable beyond TNBC (Table S1).

### 2.2. Epacadostat Significantly Reduces the Levels of the PARP Substrate NAD^+^ in MDA-MB-468 Cells

**Figure 2:**
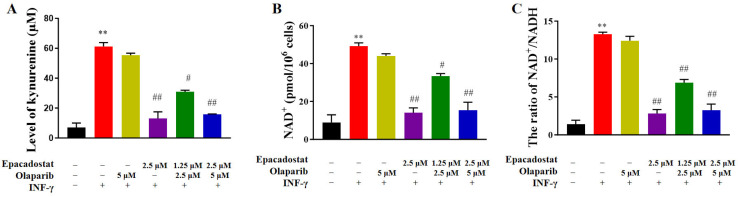
Epacadostat efficiently suppressed kynurenine and NAD^+^ in MDA-MB-468 cells. All cells were pre-treated with IFN—γ (50 ng/mL) for 24 h. Subsequently, they were treated with Olaparib and/or Epacadostat for 7 days. (**A**) Concentrations of kynurenine were detected by colorimetry. (**B**) Concentrations of NAD^+^ were detected by WST-8. (**C**) The ratio of NAD^+^ to NADH was calculated. All data are presented as means ± SD (*n* = 3). “+” means the compound was added and “-” means the compound was not added. Statistical significance of differences was determined by one-way ANOVA. ** *p* < 0.01 compared with control group, ^##^ *p* < 0.01 and ^#^ *p* < 0.05 compared with the IFN-γ (50 ng/mL) group.

The IDO1-kynurenine pathway has been demonstrated to serve as a source of NAD^+^ production. It is evident that NAD^+^ plays a pivotal role in PARP activity. Therefore, the present study sought to ascertain whether IDO1 inhibitors contribute to the reduction in NAD^+^ levels. To this end, MDA-MB-468 cells were exposed to Epacadostat, Olaparib, or a combination of the two. First, we examined the effect of these treatments on kynurenine and NAD^+^ levels in the absence of IFN-γ stimulation. The results showed that treatment with Epacadostat alone or in combination with Olaparib resulted in a substantial reduction in kynurenine and NAD^+^ levels, which is consistent with the cell viability results (Figure S4). To better amplify the IDO1-mediated metabolic effects, IFN-γ was subsequently used as a stimulus to further activate the IDO1/kynurenine pathway. As shown in Figure 2, the stimulation of IFN-γ (50 ng/mL) led to a significant increase in kynurenine levels in MDA-MB-468 cells, in comparison to the control group. Conversely, treatment with Epacadostat alone or in combination with Olaparib resulted in a substantial reduction in kynurenine levels, suggesting a substantial inhibition of IDO1 enzymatic activity (Figure 2A). Subsequent to IFN-γ stimulation, a substantial increase in both NAD^+^ levels and the NAD^+^/NADH ratio was also observed. However, treatment with Epacadostat, either as a monotherapy or in combination with Olaparib, resulted in a substantial reversal of these effects (Figure 2B,C). The findings suggest that Epacadostat can decrease intracellular NAD^+^ levels and may modulate PARP function.

### 2.3. Epacadostat and Olaparib Synergistically Promote the Generation of ROS in MDA-MB-468 Cells

**Figure 3:**
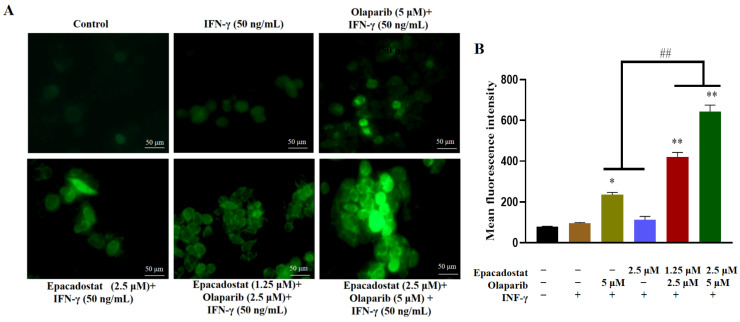
Epacadostat and/or Olaparib induce intracellular reactive oxygen species (ROS) generation through their inhibitory effects on PARP1 and IDO1. All cells were pre-treated with IFN-γ (50 ng/mL) for 24 h. Subsequently, they were treated with Olaparib and/or Epacadostat for 7 days. (**A**) ROS production was assessed by detection of DCFH fluorescence (green). Cells were also observed with phase-contrast microscopy. Scale bar, 50 μm. (**B**) Quantification of ROS fluorescence intensity in MDA-MB-468 cells treated with Epacadostat and/or Olaparib. “+” means the compound was added and “-” means the compound was not added. All data are presented as means ± SD (*n* = 3). Statistical significance of differences was determined by one-way ANOVA. ** *p* < 0.01 and * *p* < 0.05 compared with control group, ^##^ *p* < 0.01 compared with the combination group of Epacadostat and Olaparib.

PARP1/2 inhibitors have been demonstrated to activate NADPH oxidases, resulting in elevated levels of ROS. However, the knockdown of IDO1 via shRNA or treatment with IDO1 inhibitors has also been observed to elevate ROS levels and exert a significant growth-inhibitory effect on cancer cells. The present study therefore investigated the effects of Epacadostat, Olaparib, or their combination on MDA-MB-468 cells, in order to determine whether they synergistically enhance ROS production. The intracellular ROS levels were detected using the fluorescent probe 2′,7′-dichloro fluorescein diacetate (DCFH-DA), which emits green fluorescence upon oxidation. Compared with the control group, an accumulation of ROS was observed in the MDA-MB-468 cells treated with H_2_O_2_ (Figure S5). As shown in Figure 3, in comparison with single-agent treatment, the combination of Olaparib and Epacadostat resulted in a substantial increase in ROS level in MDA-MB-468 cells. The findings suggest that co-treatment with Olaparib and Epacadostat promotes ROS accumulation, thereby enhancing the sensitivity of tumor cells to DNA damage through oxidative stress.

### 2.4. Epacadostat Promotes Olaparib-Induced DNA Damage

**Figure 4:**
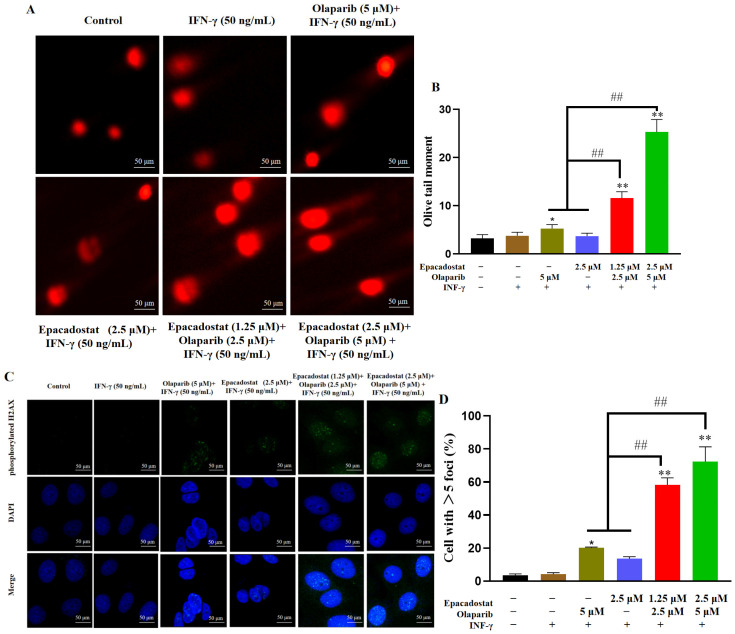
The effects of Epacadostat and/or Olaparib on DNA damage. Cells were pre-stimulated with IFN-γ (50 ng/mL) for 24 h before drug treatment. (**A**) The effects of Epacadostat and/or Olaparib on DNA damage detected by alkaline comet assay. (**B**) Quantitative analysis was conducted using the comet analysis software CASP of 1.2.3beta2 version. (**C**) The expression of phosphorylated histone H2AX was analyzed by immunofluorescence. (**D**) The accumulation of phosphorylated histone H2AX foci and the percentages of positive cells (those exhibiting more than five foci per cell) were calculated based on the analysis of approximately 200 cells. “+” means the compound was added and “-” means the compound was not added. All data are presented as means ± SD (*n* = 3). Statistical significance of differences was determined by one-way ANOVA. * *p* < 0.05 and ** *p* < 0.01 compared with control group, ^##^ *p* < 0.01 compared with the combination group of Epacadostat and Olaparib.

To further investigate the extent of DNA damage, the comet assay was conducted to evaluate the effects of Epacadostat and/or Olaparib. Typically, damaged DNA exhibits a tail in the comet assay, resembling a comet [20]. As shown in Figure 4A,B, following a 7-day treatment period, Olaparib resulted in an augmentation of the length of tail DNA in the MDA-MB-468 cell line in comparison with the control group. Notably, the combination of Olaparib and Epacadostat at lower concentrations resulted in a greater tail length compared to the monotherapy groups, indicating enhanced efficacy in inducing DNA damage. Phosphorylated H2AX localizes to sites of damaged DNA and recruits DNA repair effectors. The accumulation of phosphorylated H2AX serves as an indicator of the extent of DNA double-strand breaks. Subsequently, immunofluorescence staining was performed to analyze the expression of phosphorylated H2AX. As shown in Figure 4C,D, consistent with the outcomes of the comet assay, the combination of Epacadostat and Olaparib led to a substantial augmentation in the expression of phosphorylated H2AX in vitro at reduced concentrations when compared to the monotherapy. The findings indicate that the combination of Epacadostat and Olaparib induces DNA damage synergistically.

### 2.5. Olaparib and Epacadostat Synergistically Promote Cell Apoptosis by Increasing DNA Damage in MDA-MB-468 Cells

**Figure 5:**
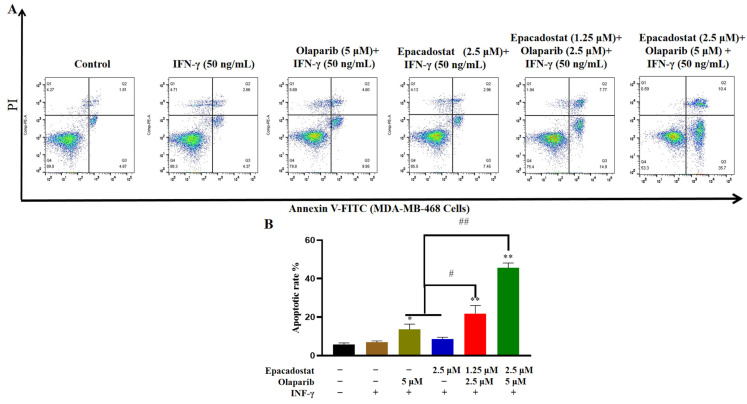
Epacadostat and/or Olaparib induces apoptosis of MDA-MB-468 cells. All cells were pre-treated with IFN-γ (50 ng/mL) for 24 h before exposure to the drugs. (**A**). The apoptosis rate of MDA-MB-468 cells was measured by flow cytometry. (**B**). Apoptosis rate in each group was analyzed using Graphpad 8.0 software. All data are presented as means ± SD (*n* = 3). “+” means the compound was added and “-” means the compound was not added. Statistical significance of differences was determined by one-way ANOVA. ** *p* < 0.01 and * *p* < 0.05 compared with control group, ^##^ *p* < 0.01 and ^#^ *p* < 0.05 compared with the combination group of Epacadostat and Olaparib.

Severe DNA damage that remains unrepaired across multiple cell cycles has been confirmed to lead to apoptosis. We first measured intracellular NAD^+^ levels at an early time point, prior to the onset of significant apoptotic cell death. The results showed that treatment with Epacadostat alone or in combination with Olaparib significantly reduced intracellular NAD^+^ levels at 48 h (Figure S6). The process of apoptosis was subsequently evaluated in MDA-MB-468 cells using flow cytometry with Annexin V staining on the 7th day. As shown in Figure 5, the results indicated that single-agent treatment led to a modest increase in the proportion of apoptotic MDA-MB-468 cells. However, the combination of Epacadostat and Olaparib resulted in a substantial augmentation of apoptosis, which exhibited a concentration-dependent manner. Consequently, the combination of Epacadostat and Olaparib exhibited a synergistic effect, promoting apoptosis in MDA-MB-468 cells.

### 2.6. Olaparib and Epacadostat Synergistically Increased Cell Cycle Arrest

**Figure 6:**
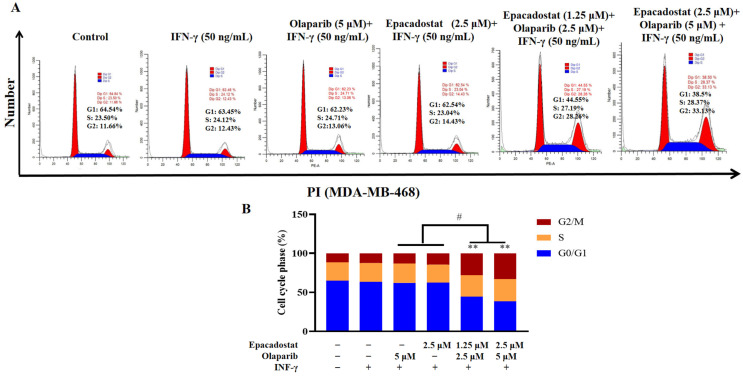
The effects of Epacadostat and/or Olaparib on cell cycle progression. All cells were pre-treated with IFN-γ (50 ng/mL) for 24 h before exposure to the drugs. (**A**) Cell cycle distribution of MDA-MB-468 cells was measured by flow cytometry using propidium iodide (PI) staining. (**B**) Quantitative measurement of cell populations at each cell cycle phase. “+” means the compound was added and “-” means the compound was not added. All data are presented as means ± SD (*n* = 3). Statistical significance of differences was determined by one-way ANOVA. ** *p* < 0.01 compared with control group, ^#^ *p* < 0.05 compared with the combination group of Epacadostat and Olaparib.

When DNA is damaged, the cell pauses the cell cycle to conduct DNA repair. The cell cycle was evaluated in MDA-MB-468 cells using flow cytometry with propidium iodide (PI) staining. As shown in Figure 6, the results indicated that Olaparib or Epacadostat induced a slight cell cycle arrest at the S and G2/M phases in MDA-MB-468 cells. Notably, the combination of Epacadostat and Olaparib significantly inhibited the S and G2/M transitions, resulting in cell cycle arrest at these phases. Therefore, the combination of Epacadostat and Olaparib synergistically enhanced DNA damage in MDA-MB-468 cells by inducing cell cycle arrest in the S and G2/M phases.

### 2.7. Olaparib and Epacadostat Synergistically Promote DNA Damage by Inhibiting Expression of BRCA1 and RAD51 in the HR Signaling Pathway

**Figure 7:**
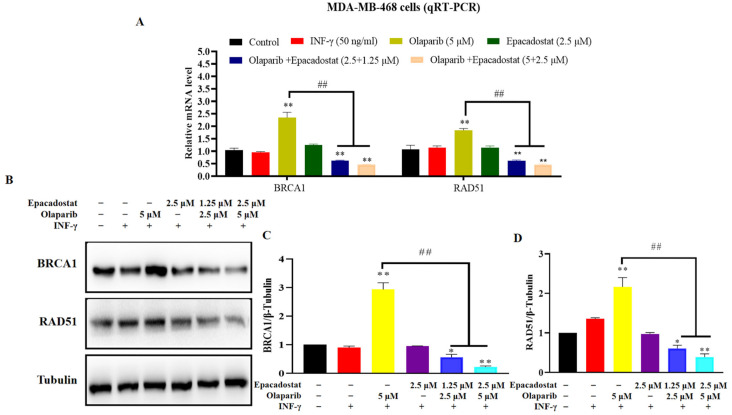
The effects of Epacadostat and/or Olaparib on the expression of BRCA1 and RAD51 in vitro. Cells were pre-stimulated with IFN-γ (50 ng/mL) for 24 h to induce the over-expression of IDO1 before treatment. (**A**) Quantitative PCR analysis of BRCA1 and RAD51 mRNA levels following the indicated treatments. Data were normalized to TBP and expressed as fold change relative to the control group. (**B**) Western blot analysis of BRCA1 and RAD51 proteins in the tumor cells treated with Epacadostat and/or Olaparib. (**C**) The protein levels BRCA1 was quantified by ImageJ (ImageJ, RRID: SCR_003070) and normalized to the β-tubulin protein content. (**D**) The protein levels RAD51 was quantified by ImageJ and normalized to the β-tubulin protein content. “+” means the compound was added and “-” means the compound was not added. All data are presented as the mean ± SD of three independent experiments. Statistical significance was determined using one-way ANOVA. * *p* < 0.05 and ** *p* < 0.01 compared with control group, ^##^ *p* < 0.01 compared with the combination group of Epacadostat and Olaparib.

It has been established that HR repair is a critical pathway for the repair of DNA damage caused by PARP inhibitors. The present study was conducted to investigate whether the inhibitory effects of Epacadostat or Olaparib, either alone or in combination, on MDA-MB-468 cells are associated with the HR repair signaling pathway. First, the mRNA expression levels of BRCA1 and RAD51 were assessed through qRT-PCR analysis. In addition, six DNA-unrelated genes (ARF1, RAB7A, CTSD, CLTC, SQSTM1 and TFRC) were also detected as specificity controls (Figure S7). As demonstrated in Figure 7, treatment with Olaparib for 7 days only resulted in a significant upregulation of both BRCA1 and RAD51 mRNA expression, indicating transcriptional activation of HR repair mechanisms (Figure S3). However, the combination of Epacadostat and Olaparib exhibited substantial suppression of this upregulation, suggesting that the combination treatment specifically downregulates genes involved in HR repair (Figure 7A). Meanwhile, Western blot analysis revealed that the protein levels of BRCA1 and RAD51 were significantly decreased following combined treatment compared to either drug alone, which was consistent with the mRNA expression results (Figure 7B–D). More importantly, the formation of RAD51 foci serves as an accurate marker of HR, which further validates our results (Figure S8). Therefore, the results suggest that Olaparib and Epacadostat synergistically promote DNA damage by inhibiting the expression of BRCA1 and RAD51 in the HR signaling pathway in vitro.

### 2.8. Epacadostat and Olaparib Synergistically Inhibit the Growth of MDA-MB-468 Cells in Tumor-Bearing Mice

**Figure 8:**
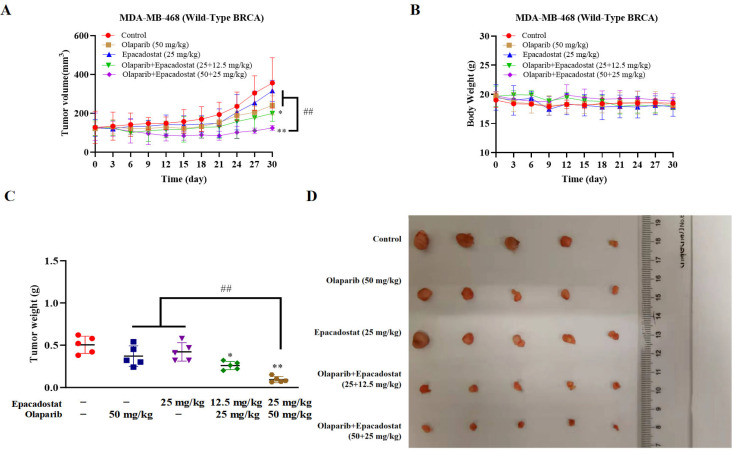

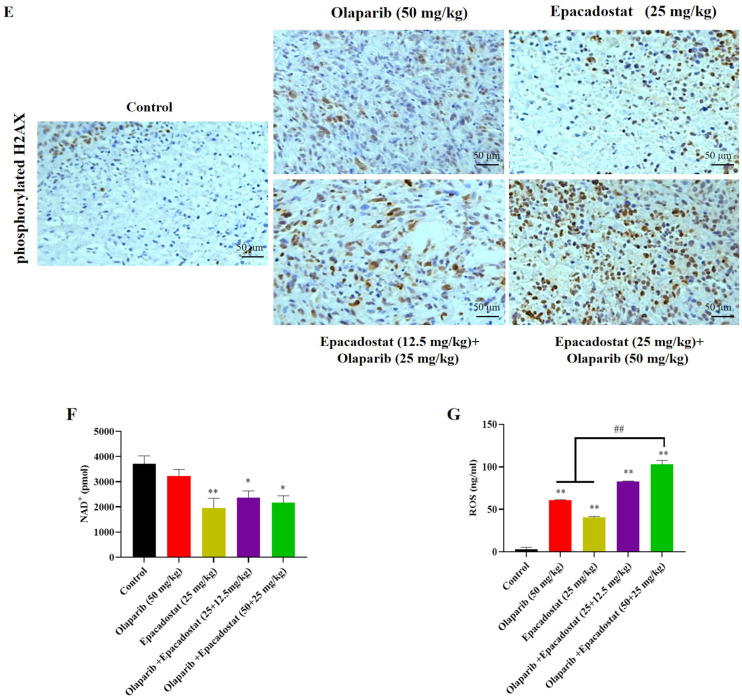
Epacadostat sensitizes MDA-MB-468 cells to Olaparib in vivo. All in vivo treatments were performed without exogenous IFN-γ supplementation. (**A**) Tumor growth curves after treatment with Epacadostat and/or Olaparib in the MDA-MB-468 xenografted model. (**B**) Body weight after treatment with Epacadostat and/or Olaparib in the MDA-MB-468 xenografted model. (**C**) Tumor weight. (**D**) Removal, collection and photographing of the tumor. (**E**) Immunohistochemical staining of phosphorylated H2AX. (**F**) Concentrations of NAD^+^ were detected by WST-8 in tumor tissue. (**G**) Concentrations of ROS were detected by a DCF kit. All data are presented as means ± SD (*n* = 5). Statistical significance of differences was determined by one-way ANOVA. * *p* < 0.05 and ** *p* < 0.01 compared with control group, ^##^ *p* < 0.01 compared with the combination group of Epacadostat and Olaparib.

The anti-tumor activity of Epacadostat or Olaparib, either as a monotherapy or in combination, was evaluated in an MDA-MB-468 xenograft mouse model. When the tumor volume reached 50–80 mm^3^, the Balb/c nude mice were treated with normal saline (NS), Olaparib (50 mg/kg), Epacadostat (25 mg/kg), a combination of Olaparib (25 mg/kg) and Epacadostat (12.5 mg/kg), or a combination of Olaparib (50 mg/kg) and Epacadostat (25 mg/kg) via intraperitoneal injection for 30 days. As demonstrated in Figure 8A–D, the size and weight of tumors were inhibited by Olaparib (50 mg/kg) or Epacadostat (25 mg/kg) in comparison to the control group. It is noteworthy that the combination of Epacadostat and Olaparib resulted in a greater inhibition of tumor growth in a dose-dependent manner compared to the individual monotherapies. No significant loss of body weight was observed in any treatment group, suggesting a favourable safety profile. Subsequently, immunohistochemical staining of tumor tissues was performed to assess the extent of DNA damage. As demonstrated in Figure 8E, the expression level of phosphorylated H2AX was significantly increased following combination treatment, indicating enhanced DNA damage. To further investigate whether the synergistic effect of Epacadostat and Olaparib arises from a reduction in NAD^+^ production or from an increase in ROS levels, the levels of NAD^+^ and ROS in tumor tissues were measured using ELISA and WST-8 assays, respectively. As demonstrated in Figure 8F,G, there was a decline in NAD^+^ levels in both the Epacadostat group and the combination group of Epacadostat and olaparib. Moreover, the combination treatment resulted in a substantial increase in ROS levels in comparison with the levels observed in response to either agent administered individually. The findings suggest that Epacadostat and Olaparib may exert synergistic anti-tumor effects in vivo by simultaneously depleting NAD^+^ and elevating ROS levels.

### 2.9. Epacadostat and Olaparib Synergistically Inhibits Tumor Growth on MDA-MB-468 Tumor Xenograft Involved in BRCA1 and RAD51 Expression

**Figure 9:**
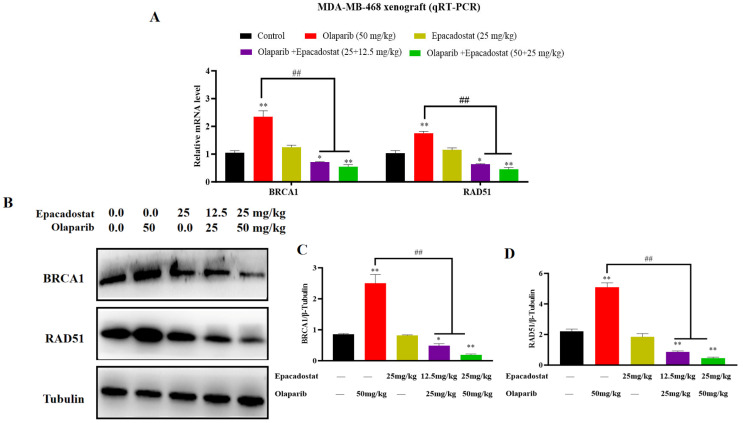
The effect of Epacadostat and/or Olaparib on the expression of BRCA1 and RAD51 in vivo. (**A**) Quantitative PCR analysis of BRCA1 and RAD51 mRNA levels following the indicated treatments. Data were normalized to GAPDH and expressed as fold change relative to the control group. (**B**) Western blot analysis of BRCA1 and RAD51 proteins in the tumor tissues of mice treated with Epacadostat and/or Olaparib. (**C**) The protein levels of BRCA1 were quantified by ImageJ and normalized to the β-tubulin protein content. (**D**) The protein levels of RAD51 were quantified by ImageJ and normalized to the β-tubulin protein content. All data are presented as the mean ± SD of three independent experiments. Statistical significance was determined using one-way ANOVA. * *p* < 0.05 and ** *p* < 0.01 compared with control group, ^##^ *p* < 0.01 compared with the combination group of Epacadostat and Olaparib.

To explore the mechanism underlying the synergistic inhibitory effects of Epacadostat and Olaparib on tumor growth in vivo, a qRT-PCR assay was performed. The present study therefore investigated the effects of Epacadostat, Olaparib, and their combination on the mRNA expression of BRCA1 and RAD51 in the MDA-MB-468 xenograft model. As demonstrated in Figure 9A, the results indicated that Olaparib-induced mRNA expression of BRCA1 and RAD51 could promote HR repair in vitro. However, the combination of Epacadostat and Olaparib has been shown to significantly inhibit the mRNA expression of BRCA1 and RAD51 in a dose-dependent manner. Further, Western blot analysis revealed that the protein levels of BRCA1 and RAD51 were also substantially reduced in the combination group compared with either monotherapy, consistent with the mRNA expression results (Figure 9B–D). In conclusion, the results indicate that the combination of Epacadostat and Olaparib produces a synergistic anti-tumor effect, achieved through the inhibition of BRCA1 and RAD51 expression.

## 3. Discussion

TNBC remains the most challenging subtype of breast cancer, characterized by the poorest prognosis and the lowest median survival rate after relapse [21]. The utilization of synthetic lethality as a targeting strategy for PARP1/2 has emerged as a promising approach for patients diagnosed with TNBC who carry mutated BRCA1/2 genes. BRCA1/2 and RAD51 proteins are critical participants in the process of DNA precision repair. The presence of wild-type BRCA1/2 would result in the weakening of the repression of DNA damage repair by PARP1/2 inhibitors, and the majority of TNBC cases are of the BRCA1/2 wild-type [22,23]. A Phase II study showed that the objective response rates to PARP1/2 inhibitors were 55.6% in BRCA-mutated TNBC patients and 7.1% in BRCA-proficient patients, who constitute the majority of TNBC cases [24]. Therefore, enhancing the sensitization of PAPR1/2 inhibitors has become an urgent clinical need. Recent studies have further demonstrated that combining PARP1/2 inhibitors with immune checkpoint inhibitors, antiangiogenic agents, and various small-molecule inhibitors can overcome drug resistance. However, the efficiency and overlapping toxicity of these combinations vary among different cancers, which limits their clinical application [25].

IDO1, as a critical catalytic enzyme for L-tryptophan metabolism, plays a role in inhibiting immune cell cytotoxicity against tumor cells [26]. A novel function of IDO1 has also been revealed, namely its ability to promote resistance of human tumor cells to PARP1/2 inhibitors, a property which is independent of its immune activity [12]. We found that the expression level of IDO1 is elevated in cases of breast cancer. In this study, we investigated the growth-inhibitory effects of a combination strategy involving an IDO1 inhibitor Epacadostat and a PARP1/2 inhibitor Olaparib on BRCA-proficient (HRR-proficient) TNBC cells.

We found that the combined treatment with Olaparib and Epacadostat was more effective in inhibiting the growth of TNBC cells and significantly increased the rate of cell apoptosis compared to treatment with either drug alone. It indicated that the addition of IDO1 inhibitors significantly increased the sensitivity of tumor cells to Olaparib, which was consistent with the description by Maleki, Vareki et al. [12]. We proposed that the ability of Epacadostat to sensitize BRCA-proficient TNBC cells to Olaparib might be attributed to the following mechanisms. Firstly, the markedly reduced intracellular NAD^+^ levels caused by Epacadostat impaired the DNA damage repair (Figure 2A–C and Figure 8F). NAD^+^ is the substrate for the synthesis of poly (ADP-ribose) (PAR), which is catalysed by poly (ADP-ribose) polymerase 1/2 (PARP1/2). This reaction is stimulated in the presence of DNA damage. PAR is then covalently attached to PARP1/2 in a process known as PARylation, which weakens the affinity of PARP1/2 for DNA and promotes its dissociation from DNA nicks. This initiates the recruited DNA repair enzyme to repair the DNA damage [27]. We speculate that depleting NAD^+^ will impair PARP-mediated DNA repair, thereby enhancing the cytotoxic effects of olaparib by inhibiting PARP1/2. Secondly, Epacadostat and Olaparib synergistically increased ROS accumulation (Figure 3 and Figure 8G), resulting in exacerbated DNA damage. PARP1/2 inhibitors have been reported to activate NADPH oxidases, thereby promoting ROS production [28]. Knockdown of IDO1 decreased the generation of NAD^+^ while increasing the generation of ROS [29]. ROS are potent inducers of oxidative stress that can lead to various types of irreparable DNA damage. As demonstrated by the comet assay results in this study, DNA damage was exacerbated. Immunofluorescence results, showing the phosphorylated H2AX foci (a sensitive marker of DNA double-strand breaks) per nucleus, further confirmed an increased number of DNA lesions and an impaired cellular repair ability. ROS induce DNA damage that can be detected using γH2AX and comet assays. N-acetylcysteine (NAC) significantly reduces ROS levels and alleviates ROS-induced DNA damage in various cell types [30]. However, the relative contributions of oxidative stress and NAD^+^ depletion to the observed synergistic DNA damage remain to be clarified. Thirdly, the combined treatment of Epacadostat and Olaparib significantly suppressed BRCA1 and RAD51 expression at both the transcriptional and protein levels. At the transcriptional level, Epacadostat and Olaparib markedly reduced BRCA1 and RAD51 mRNA expression (Figure 7A and Figure 9A), indicating that the downregulation of these HR genes may contribute to the impaired DNA repair capacity. At the protein level, this suppression was also evident in vitro and in vivo (Figure 7B and Figure 9B), further confirming that the combined treatment weakened HR repair capacity. BRCA1 and RAD51 are key mediators of HRR and are recruited to damaged DNA to facilitate the precise repair of double-strand breaks. Interestingly, Olaparib alone induced a compensatory upregulation of BRCA1 and RAD51 expression, which may represent an adaptive resistance mechanism to enhance HRR capacity. In contrast, Epacadostat co-treatment abrogated this compensatory response, thereby sensitizing BRCA-proficient TNBC cells to Olaparib. Previous studies have shown that RAD51 mutations increase sensitivity to PARP inhibitors, whereas exogenous overexpression of RAD51 restores HR function and reduces Olaparib sensitivity [31]. Future plans include further research on the mechanisms of DNA HR.

Although we have elucidated the synergistic sensitization effects and underlying mechanisms of Epacadostat and Olaparib in TNBC cells both in vitro and in vivo, HRR is a high-fidelity DNA repair mechanism requiring the sequential activities of a series of proteins [32,33,34]. Furthermore, our data demonstrate a significant reduction in BRCA1 and RAD51 expression levels, as well as the absence of RAD51 foci formation following combination treatment; however, these findings do not necessarily indicate complete HR deficiency. Future studies employing functional recombination tests (such as with HR test substrates from the Maria Jasin lab [35]) or combining RAD51 foci analysis with cell-cycle markers (EdU incorporation or geminin expression) are warranted to definitively determine the functional status of HR under this dual inhibition strategy. Moreover, there are multiple effective IDO1 and PARP1/2 inhibitors available, but only Epacadostat and Olaparib were selected for mechanistic investigation in this study. Therefore, the mechanisms underlying the dual inhibition of IDO1 and PARP1/2 in the DNA damage response need further validation using additional inhibitors targeting these pathways.

## 4. Materials and Methods

### 4.1. Reagents and Antibodies

Epacadostat, Olaparib, IFN-γ, bovine serum albumin (BSA), and IL-15 cell culture medium were obtained from Sigma-Aldrich (St. Louis, MO, USA). The NAD^+^ detection kit, reactive oxygen species (ROS) detection kit, Annexin V-FITC apoptosis detection kit, MTT cell proliferation assay kit, comet assay kit, 4,6-diamidino-2-phenylindole (DAPI), and crystal violet were purchased from KeyGEN Biotech (KeyGEN BioTECH, Nanjing, China). BRCA1 Antibody (1:1000, #9010), RAD51 (D4B10) Rabbit mAb (1:1000, #8875), anti-mouse IgG-HRP-linked antibody (1:3000, #7076), anti-rabbit IgG -HRP-linked antibody (1:3000, #7074), and anti-β-tubulin (8H10D10) mouse mAb (1:1000, #3700) were purchased from Cell Signaling Technology (Boston, MA, USA). Alexa Fluor 488 Anti-Human phospho-Histone H2AX (Ser139) mouse mAb (1:100, P16104) was purchased from Affymetrix ebioscience (Santiago, CA, USA). All other chemicals used were of analytical grade and used without further purification unless otherwise specified. Epacadostat and Olaparib were dissolved in DMSO to prepare 10 mM stock solutions, with gentle agitation until fully dissolved. Aliquots of the stock solutions were stored at −4 °C.

### 4.2. Cell Culture

The BRCA-proficient TNBC cell lines MDA-MB-231 and MDA-MB-468 were obtained from the Cell Resource Center of the Shanghai Academy of Life Sciences (Shanghai, China). These cell lines correspond to the following ATCC reference strains (ATCC, Manassas, VA, USA): MDA-MB-231 (ATCC^®^ HTB-26™; triple-negative, ER−/PR−/HER2−) and MDA-MB-468 (ATCC^®^ HTB-132™; triple-negative, ER−/PR−/HER2−). MDA-MB-231 and MDA-MB-468 cells were cultured in sterile Leibovitz’s L-15 medium (KeyGEN Biotech, Nanjing, China) supplemented with 10% fetal bovine serum (FBS) and 1% penicillin-streptomycin. All cell lines were maintained in a humidified incubator at 37 °C with 5% CO_2_. The cells were seeded at a density of 1000–3000 cells per well, and they reached 80–90% confluence by the end of the experiment.

### 4.3. Drug-Combination Assay

The selected concentration ratios of Epacadostat to Olaparib were 1:1, 1:2, and 1:4. After a 7-day treatment of TNBC cells with combinations at these concentration ratios, the viability of MDA-MB-231 and MDA-MB-468 cells was assessed using the CCK-8 assay. Untreated cells (or vehicle control, 0.1% DMSO) served as a control, and the IC_50_ values were calculated using GraphPad Prism version 8.0. The combination index (CI) was calculated using the formula CI = CE, X/ICX, E + CO, X/ICX, O [36]. In this formula, CE, X and CO, X represent the concentrations of Epacadostat and Olaparib that achieve X% inhibition rate in combination, respectively. ICX, E and ICX, O represent the concentrations of the single agents (Epacadostat or Olaparib) that achieve the same level of growth inhibition rate. A combination index (CI) of less than 1.0 indicates synergism, a CI equal to 1.0 indicates additivity, and a CI of more than 1.0 indicates antagonism.

### 4.4. Colony Formation Assay

MDA-MB-468 cells were seeded in 6-well plates at a density of 1 × 10^3^ cells per well and allowed to adhere overnight. Cells were then treated with Epacadostat (2.5 μM), Olaparib (5 μM), a combination of Olaparib (2.5 μM) and Epacadostat (5 μM), or a combination of Olaparib (1.25 μM) and Epacadostat (2.5 μM) for 14 days, with the medium replaced every 3–4 days. At the end of the incubation period, colonies were fixed with 4% paraformaldehyde for 15 min at room temperature, stained with 0.1% crystal violet for 30 min, rinsed gently with distilled water, and air-dried. Colony images were captured using an Olympus BX53 inverted microscope (Olympus Corporation, Tokyo, Japan).

### 4.5. Comet Assay

After 24 h of incubation with IFN-γ (50 ng/mL), the cells were treated with Epacadostat (2.5 μM), Olaparib (5 μM), a combination of Olaparib (2.5 μM) and Epacadostat (5 μM), or a combination of Olaparib (1.25 μM) and Epacadostat (2.5 μM) for 7 days. The duration of 7 days was also determined by a previous literature report [5]. Untreated cells (or vehicle control, 0.1% DMSO) served as a control. Alkaline comet assay was performed with a commercially available kit according to the manufacturer’s protocol. Briefly, cells at a density of 1 × 10^4^ cells/mL were mixed with low-melting agarose at a 1:10 (*v*/*v*) ratio, layered onto precoated microscope slides (with 1% normal melting agarose as the base layer), and lysed in lysis buffer for 2 h at 4 °C. Following electrophoresis at 21 V for 30 min in 1× Tris-acetate-EDTA buffer, the cells were stained with propidium iodide (PI, 5 μg/mL) for 15 min in the dark and examined under an inverted fluorescence microscope. Five random fields were captured from each slide for analysis.

### 4.6. Analysis of Apoptosis and Cycle Assays

Apoptosis was detected using Annexin V-FITC/PI apoptosis detection kits (KeyGEN Biotech, Nanjing, China). The cell cycle was analyzed by a PI cell cycle detection kit (KeyGEN Biotech, Nanjing, China). MDA-MB-468 cells were seeded in 6-well plates at a density of 2 × 10^5^ cells/well. All cells (including the control group) were first incubated with IFN-γ (50 ng/mL) for 24 h, followed by treatment with Epacadostat (2.5 μM), Olaparib (5 μM), a combination of Olaparib (2.5 μM) and Epacadostat (5 μM), or a combination of Olaparib (1.25 μM) and Epacadostat (2.5 μM) for 7 days. Following the manufacturer’s protocol, cells were collected and stained with Annexin V-FITC and PI for 15 min at room temperature in the dark to analyze their apoptotic state. Apoptosis was assessed using flow cytometry (FACSCelesta flow cytometer; BD Biosciences, San Jose, CA, USA), and apoptosis rates, including early apoptosis (Annexin V^+^/PI^−^) and late apoptosis (Annexin V^+^/PI^+^), were quantified with FlowJo software (version 7.6).

### 4.7. Immunofluorescence Assay

After 24 h of incubation with IFN-γ (50 ng/mL), the cells were treated with Epacadostat (2.5 μM), Olaparib (5 μM), a combination of Olaparib (2.5 μM) and Epacadostat (5 μM), or a combination of Olaparib (1.25 μM) and Epacadostat (2.5 μM) for 7 days. After treatment, the cells were fixed with 4% paraformaldehyde for 15 min at room temperature and permeabilized with PBS containing 0.2% (*v*/*v*) Triton X-100 for 10 min. Subsequently, they were blocked with PBS containing 1% (*w*/*v*) BSA for 1 h at room temperature, followed by three washes with PBS (5 min each). Cells were then stained with an Alexa Fluor 488-labeled anti-phosphorylated histone 2AX (γH2AX) antibody (1:100, Affymetrix eBioscience, San Diego, CA) overnight at 4 °C. After three washes with PBS, DAPI was used to counterstain cell nuclei for 10 min at room temperature, followed by two washes with PBS. The fluorescence signals were observed using a Carl Zeiss LSM700 laser scanning confocal microscope (Carl Zeiss, Jena, Germany). Five random fields per sample were analyzed for quantification.

### 4.8. Determination of ROS Levels

MDA-MB-468 cells were seeded in 6-well plates at a density of 2 × 10^5^ cells/mL. After 24 h of incubation with IFN-γ (50 ng/mL), the cells were treated with Epacadostat (2.5 μM), Olaparib (5 μM), a combination of Olaparib (2.5 μM) and Epacadostat (5 μM), or a combination of Olaparib (1.25 μM) and Epacadostat (2.5 μM) for 7 days. Subsequently, the cells were stained with 10 μM DCFH-DA (Beyotime Biotech, Nanjing, China) for 30 min at 37 °C and washed twice with serum-free medium for 5 min each. For in vitro fluorescence microscopy, images were captured using the EVOS FL Imaging System (Thermo Fisher Scientific, Waltham, MA, USA) at 20× magnification with excitation at 488 nm and emission at 525 nm. For in vivo quantification of ROS levels in tumor tissues collected from MDA-MB-468 xenograft models, a commercial ROS detection kit (Beyotime Biotech, Nanjing, China) was used according to the manufacturer’s instructions.

### 4.9. Determination of NAD^+^, and NAD^+^/NADH Levels

After a 7-day treatment with Epacadostat, Olaparib, or their combination in the presence of IFN-γ (50 ng/mL), MDA-MB-468 cells, seeded at 2 × 10^5^ cells per well in 6-well plates, were collected through trypsinization, washed twice with ice-cold PBS, and resuspended in 200 μL of NAD^+^/NADH extraction buffer. The cells were lysed on ice for 15 min with occasional vortexing. The lysates were then centrifuged at 12,000× *g* for 5 min at 4 °C to remove cell debris, and the supernatants were collected for subsequent analysis. Fresh tumor tissues (approximately 50 mg) from xenograft models were weighed and homogenized in 500 μL of ice-cold NAD^+^/NADH extraction buffer (1:10, *w*/*v*) using a TissueLyser II homogenizer (Qiagen, Hilden, Germany) at 50 Hz for 2 min, followed by a centrifugation at 12,000× *g* for 10 min at 4 °C to collect the supernatants. The NAD^+^ and NAD^+^/NADH ratios were determined using an NAD^+^/NADH assay kit (Beyotime Institute of Biotechnology, Shanghai, China) following the manufacturer’s protocols. Briefly, cells were lysed using the NAD^+^/NADH extraction buffer and centrifuged at 12,000× *g* for 5 min at 4 °C to collect the supernatants. The levels of NAD^+^ and NADH in the supernatants (100 μg/well) were analyzed and expressed as a percentage of the control group.

### 4.10. Determination of Kynurenine Levels

MDA-MB-468 cells were plated at a density of 1000 cells per well in a 96-well plate and treated with IFN-γ (50 ng/mL), Epacadostat, Olaparib, or their combination for 7 days. Following the incubation period, 140 μL of cell culture supernatant was mixed with 10 μL of 6.1 mol/L trichloroacetic acid (TCA) followed by incubation at 50 °C for 30 min. The resulting mixture underwent centrifugation at 400 rpm for 20 min to isolate the supernatant, from which 100 μL was then mixed with an equal volume of ice-cold 2% acetic acid containing p-DMAB. After shaking for 10 min under light protection conditions, absorbance measurements were conducted at 492 nM using a Thermo plate reader. Kynurenine concentrations were determined by fitting a dose–response curve with GraphPad Prism 5 software.

### 4.11. Animal Experiments Xenograft Models

All animal experiments were conducted in accordance with the guidelines for the care and use of laboratory animals and were approved by the Animal Experiment Ethics Committee of Jiangsu College of Medicine (Permit Number: XMLL2025084), Yancheng Municipal Science and Technology Commission (Permit Number: YCBK2024008). Female BALB/c nude mice (6–8 weeks old, body weight (BW) of 18–20 g) were provided by the Model Animal Research Institute of Nanjing University (Nanjing, China). MDA-MB-468 cells (1 × 10^6^ in 100 μL sterile PBS) were subcutaneously inoculated into the right flank of female BALB/c nude mice. Tumor volume was measured twice weekly using a digital caliper by a blinded observer. Tumor volume was estimated using the formula (a × b^2^)/2, where a and b represent tumor length and width, respectively. After the average tumor volume reached 50–80 mm^3^, the tumor-bearing mice were randomly divided into five groups (n = 5 per group), including a vehicle control group (administration with 0.1% DMSO in saline via intraperitoneal injection), Epacadostat group (administration with 25 mg/kg Epacadostat via intraperitoneal injection), Olaparib group (administration with 50 mg/kg Olaparib via intraperitoneal injection), and a combination group of Epacadostat and Olaparib (administration with 12.5 mg/kg Epacadostat and 25 mg/kg Olaparib via intraperitoneal injection) and a combination group of Epacadostat and Olaparib (administration with 25 mg/kg Epacadostat and 50 mg/kg Olaparib via intraperitoneal injection). No exogenous IFN-γ was administered to the animals during the xenograft experiments, as IDO1 expression in MDA-MB-468 tumors was sufficient for in vivo evaluation. After 30 days of treatment, the mice were sacrificed promptly by cervical vertebra dislocation to assess the tumor weight in each group. Xenograft tumor tissues were frozen on dry ice and stored at −80 °C for subsequent experimental analyses.

### 4.12. Immunohistochemistry

In brief, the paraffin-embedded sections were deparaffinized in xylene and then rehydrated. The deparaffinized sections were immersed in sodium citrate buffer to retrieve antigens. Subsequently, the sections were treated with 3% hydrogen peroxide for 15 min to block endogenous peroxidase activity. After washing with PBS, the slides were incubated at 4 °C with an anti-phosphorylated H2AX antibody diluted 1:2000 in the original antibody dilution buffer (Beyotime, Shanghai, China) for 12 h. Finally, the slides were counterstained with hematoxylin (Ligen, Beijing, China) and mounted. Each group of sections was examined using a DM6B upright fluorescence microscope.

### 4.13. Western Blot

Total proteins were extracted from MDA-MB-468 cells and MDA-MB-468 tumor xenografts using RIPA cell lysis buffer (Beyotime, Shanghai, China) supplemented with a protease and phosphatase inhibitor cocktail. The lysates were collected by centrifugation at 14,000 rpm for 10 min at 4 °C. After centrifugation, the supernatant was carefully removed, and protein concentration was determined using the BCA Protein Assay Kit (Thermo Scientific, Waltham, MA, USA). Equal amounts of protein from each group were subsequently prepared for Western blot analysis. Briefly, proteins were separated by sodium dodecyl sulfate-polyacrylamide gel electrophoresis (SDS-PAGE) and then electrotransferred onto a polyvinylidene difluoride (PVDF) membrane (Millipore, Billerica, MA, USA). The membranes were blocked with 5% (*w*/*v*) non-fat dry milk and then incubated with primary antibodies, including anti-BRCA1 (1:1000), anti-RAD51 (1:1000), and anti-β-Tubulin (1:5000) from Cell Signaling Technology (Beverly, MA). After extensive washing with PBST buffer, membranes were incubated with corresponding secondary antibodies (1:2000) for detection. Protein bands were subsequently quantified using ImageJ software v1.6.0 (National Institutes of Health, Bethesda, MD, USA).

### 4.14. qRT-PCR Analysis

Total RNA was extracted from MDA-MB-468 cells and MDA-MB-468 tumor xenografts using TRIzol reagent (Vazyme, Nanjing, China) following the manufacturer’s protocol. Reverse transcription was performed using the HiScript II One-Step RT-PCR Kit (Vazyme, Nanjing, China) with 1.0 μg of total RNA incorporated into a 20 μL reaction system. For quantitative PCR, 1.0 μL of the resulting cDNA was used per reaction, with each sample run in triplicate. qRT-PCR was carried out on the QuantStudio 3 Real-Time PCR Detection System (Life Technologies, New York, NY, USA) using ChamQ SYBR Q-PCR Master Mix (Vazyme, Nanjing, China). A non-template negative control was included in each run. TATA box-binding protein (TBP) was used as the internal control for normalization of mRNA expression levels. Primer sequences are provided in Table 3.

### 4.15. Gene Expression and Correlation Analysis

The differential expression of IDO1, PARP1, and PARP2 between cancer tissues and adjacent normal tissues in TCGA project was analyzed by Gene Expression Profiling Interactive Analysis version 2 (GEPIA2) (http://gepia2.cancer-pku.cn, accessed on 1 May 2025). Box plots were utilized to assess the differential expression of PARP1, PARP2, and IDO1 between adjacent normal tissues and breast-invasive carcinoma tissues of the Genotype Tissue Expression (GTEx) database with the following settings: *p*-value cutoff  =  0.01, log2FC cutoff  =  1.0, and “match TCGA normal and GTEx data”. The relationship between PARP1 and IDO1 in breast-invasive carcinoma was additionally examined using GEPIA2 from the TCGA project.

### 4.16. Statistical Analysis

Data were analyzed using statistical software SPSS 19.0 and expressed as mean ± standard deviation (SD). Differences between treatment regimens were analyzed by two-tailed Student’s *t*-test or one-way ANOVA. A *p*-value less than 0.05 (*p* < 0.05) was considered statistically significant.

## Figures and Tables

**Table 1:** CI values for the effects of combining Epacadostat with Olaparib on the proliferation of MDA-MB-231 cells after 7 days.

Cell			MDA-MB-231 Ratio (1:1)		MDA-MB-231 Ratio (1:2)		MDA-MB-231 Ratio (1:4)	
Group	Epacadostat	Olaparib	Epacadostat	Olaparib	Epacadostat	Olaparib	Epacadostat	Olaparib
IC_50_ (μM)	33.2 ± 5.2	4.7 ± 0.3	2.35 ± 0.2 **	2.35 ± 0.2 ^#^	0.93 ± 0.03 **	1.86 ± 0.06 ^##^	1.35 ± 0.04 **	5.40 ± 0.16 ^#^
CI value	/	/	0.57 ± 0.13		0.42 ± 0.07		1.19 ± 0.43	

** *p* < 0.01 compared to Epacadostat-only-treated group, ^#^ *p* < 0.05 and ^##^ *p* < 0.01 compared to Olaparib-only-treated group.

**Table 2:** CI values for the effects of combining Epacadostat with Olaparib on the proliferation of MDA-MB-468 cells after 7 days.

Cell			MDA-MB-468 Ratio (1:1)		MDA-MB-468 Ratio (1:2)		MDA-MB-468 Ratio (1:4)	
Group	Epacadostat	Olaparib	Epacadostat	Olaparib	Epacadostat	Olaparib	Epacadostat	Olaparib
IC50 (μM)	28.3 ± 3.8	2.4 ± 0.7	1.14 ± 0.3 **	1.14 ± 0.3 ^#^	0.31 ± 0.01 **	0.62 ± 0.02 ^##^	0.84 ± 0.32 **	3.36 ± 0.71 ^##^
CI value	/	/	0.52 ± 0.22		0.27 ± 0.03		1.43 ± 0.13	

** *p* < 0.01 compared to Epacadostat-only-treated group, ^#^ *p* < 0.05 and ^##^ *p* < 0.01 compared to Olaparib-only-treated group.

**Table 3:** Primer sequences for genes in qRT-PCR.

Name	Sense (5′-3′)	Antisense (5′-3′)
BRCA1	GCTCGTGGAAGATTTCGGTGT	TCATCAATCACGGACGTATCATC
RAD51	CCTCCTCTTTAACGCCTCCTG	GGGGACAACTCCCAGACTTTTT
ARF1	CCGATCAGACCTCAAGGACAG	CCCAGGGTAGCTTGTGGGA
RAB7A	GTGTTGCTGAAGGTTATCATCCT	GCTCCTATTGTGGCTTTGTACTG
CTSD	TGCTCAAGAACTACATGGACGC	CGAAGACGACTGTGAAGCACT
CLTC	ATTCTGCCAATTCGTTTTCAGGA	GCTTTCAGTGCAATTACTTTGCT
SQSTM1	GCACCCCAATGTGATCTGC	CGCTACACAAGTCGTAGTCTGG
TFRC	ACCATTGTCATATACCCGGTTCA	CAATAGCCCAAGTAGCCAATCAT
TBP	CCTGCCGATAACTATCATCTGGC	GTTTCCACGGATGCTTTCTCG

## Data Availability

Data available on reasonable request.

## Supplementary Materials

The following supporting information can be downloaded at https://www.mdpi.com/article/10.3390/molecules31061039/s1, Figure S1: Bioinformatics analysis of IDO1, PARP1, and PARP2. Figure S2: Drug–response curves of IC_50_ values for cell viability after a series of concentrations treatment of Olaparib or Epacadostat in the TNBC measured by CCK-8 assay. Figure S3: The synergistic effects of Epacadostat and Olaparib on the proliferation of MCF10A cells Figure S4: Epacadostat efficiently suppressed kynurenine and NAD^+^ in MDA-MB-468 cells. Figure S5: H_2_O_2_ induce intracellular reactive oxygen species (ROS) generation. Figure S6: Epacadostat efficiently suppressed NAD+ in MDA-MB-468 cells. Figure S7: Quantitative PCR analysis of ARF1, RAB7A, CTSD, CLTC, SQSTM1, and TFRC mRNA levels following treatment with Epacadostat and/or Olaparib. Figure S8: The effects of Epacadostat and/or Olaparib on RAD51 foci formation. Table S1: CI values for the effects of combining Epacadostat with Olaparib on the proliferation of SW1990 cells after 7 days.

## Abbreviations

The following abbreviations are used in this manuscript:

TNBC	Triple-negative breast cancer
ROS	Reactive oxygen species
HR	Homologous recombination
IDO1	Indoleamine 2,3-dioxygenase 1
DDR	DNA damage response
SSBs	Single-strand breaks
DSBs	Double-strand breaks
BER	Base excision repair
PARP1/2	Poly (ADP-ribose) polymerase 1/2
