# Supplementary material for: Epacadostat and Olaparib Synergistically Inhibit the Growth of BRCA-Proficient Triple-Negative Breast Cancer by Suppressing the Expression of BRCA1 and RAD51
Source: Molecules. 2026 Mar 20;31(6):1039. doi: 10.3390/molecules31061039 (PMC13029597; doi:10.3390/molecules31061039)
Supplement: Supplementary file 1 [file molecules-31-01039-s001.zip › molecules-4194864-supplementary.pdf]

Supplementary Information

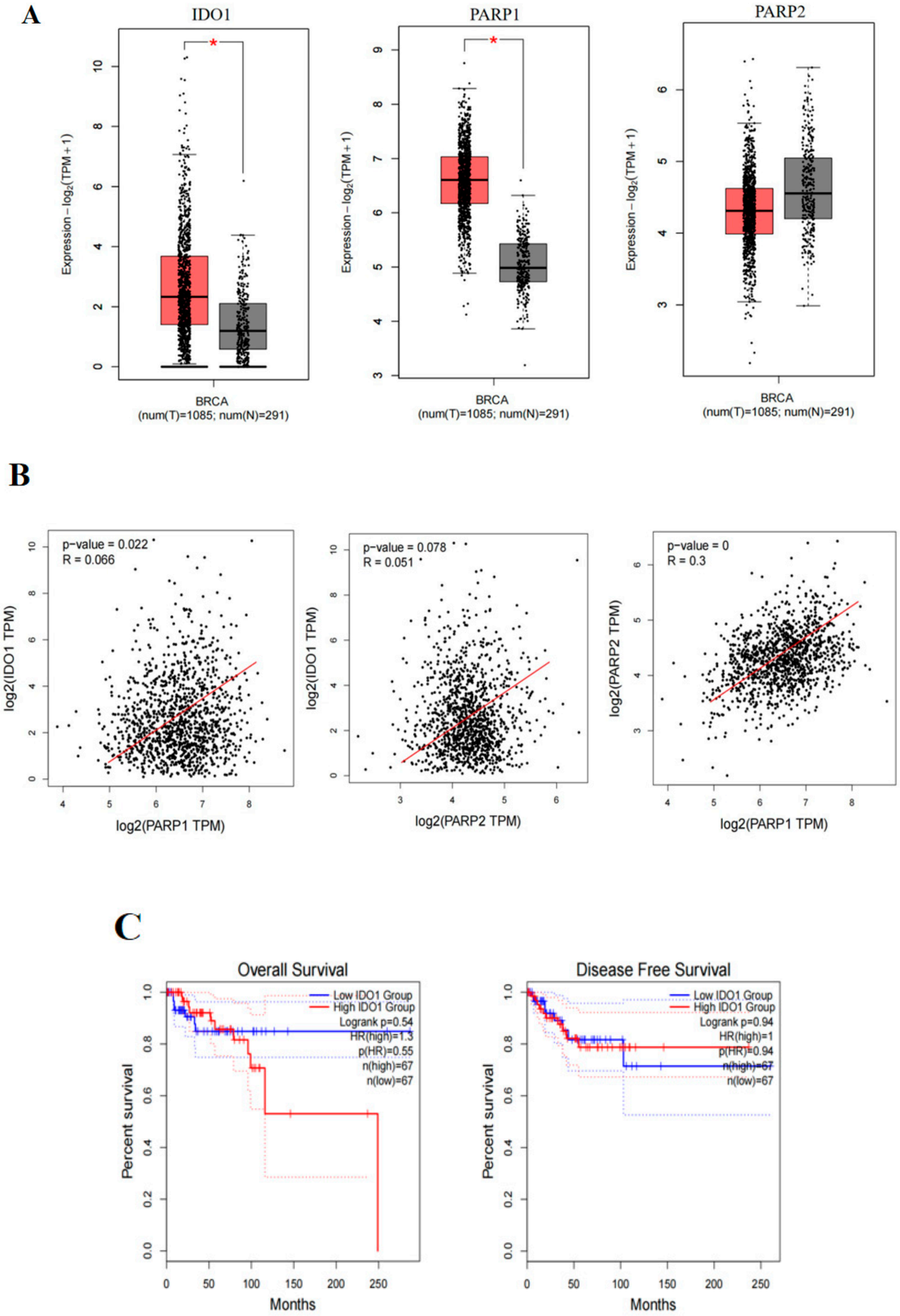

D

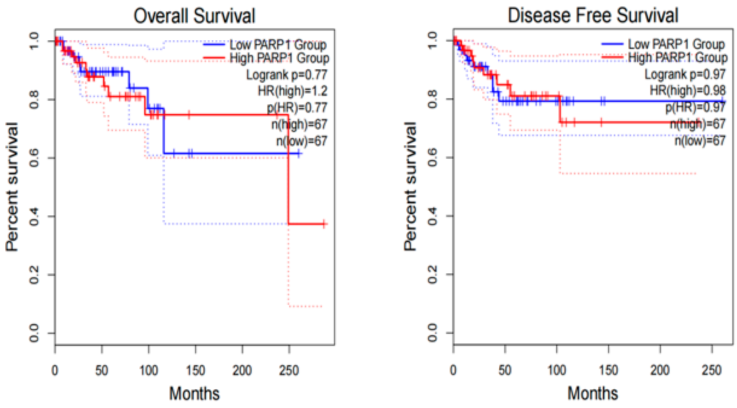

E

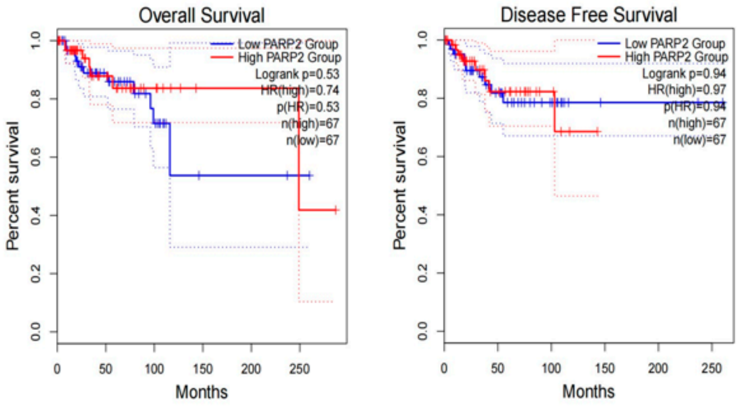

Figure S1. Bioinformatics analysis of IDO1, PARP1, and PARP2. (A) The differential expression of IDO1, PARP1 and PARP2 between breast-invasive carcinoma tissues (T) and adjacent normal tissues (N). (B) Correlation analysis of IDO1, PARP1 and PARP2 expression in breast cance. (C) Analysis of the association between patient survival and IDO1 expression. (D) Analysis of the association between patient survival and PARP1 expression. (E) Analysis of the association between patient survival and PARP2 expression.

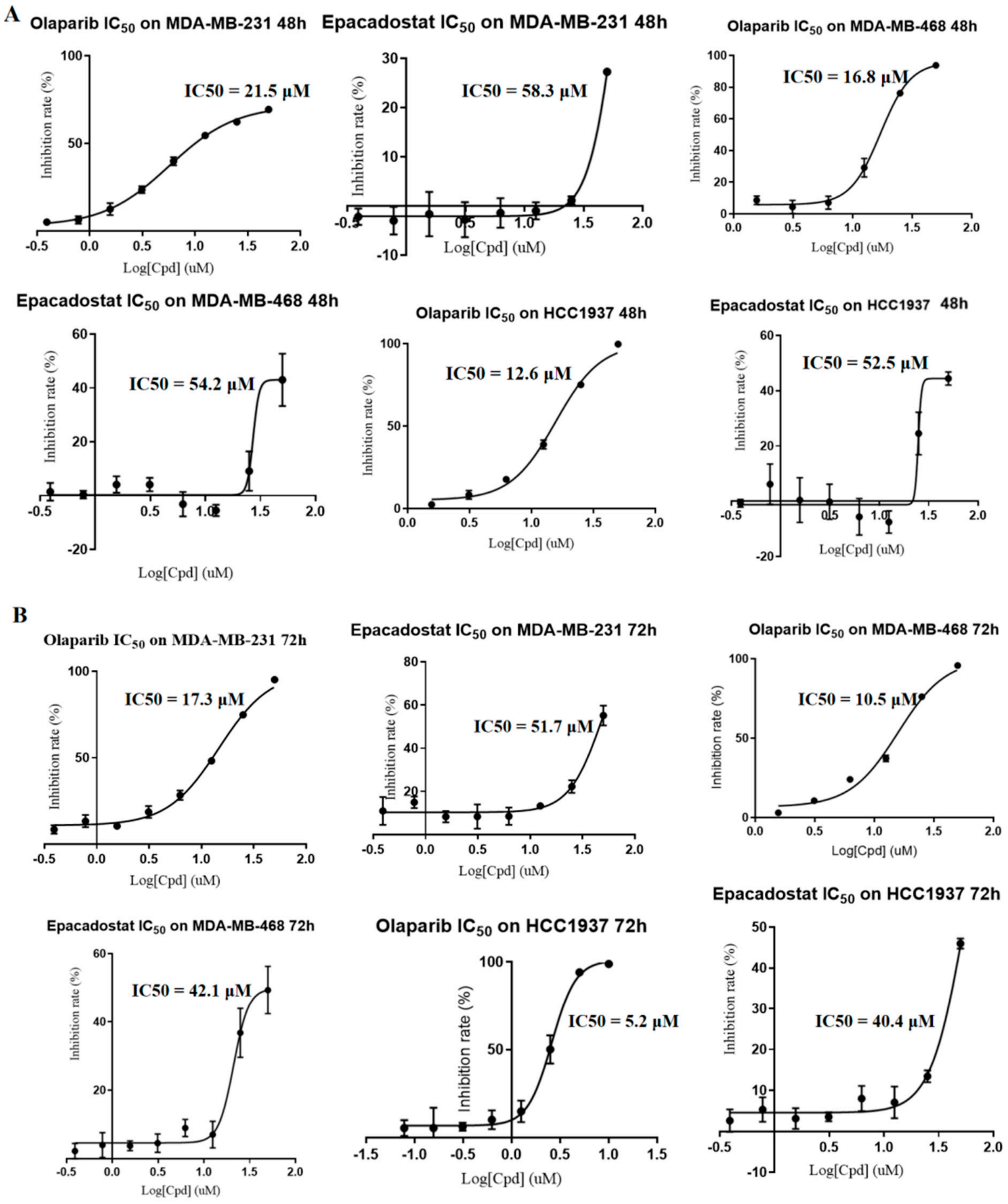
A
Olaparib IC50 on MDA-MB-231 48h
IC50 = 21.5 μM
Epacadostat IC50 on MDA-MB-231 48h
IC50 = 58.3 μM
Olaparib IC50 on MDA-MB-468 48h
IC50 = 16.8 μM
Epacadostat IC50 on MDA-MB-468 48h
IC50 = 54.2 μM
Olaparib IC50 on HCC1937 48h
IC50 = 12.6 μM
Epacadostat IC50 on HCC1937 48h
IC50 = 52.5 μM
B
Olaparib IC50 on MDA-MB-231 72h
IC50 = 17.3 μM
Epacadostat IC50 on MDA-MB-231 72h
IC50 = 51.7 μM
Olaparib IC50 on MDA-MB-468 72h
IC50 = 10.5 μM
Epacadostat IC50 on MDA-MB-468 72h
IC50 = 42.1 μM
Olaparib IC50 on HCC1937 72h
IC50 = 5.2 μM
Epacadostat IC50 on HCC1937 72h
IC50 = 40.4 μM
Inhibition rate (%)
Log[Cpd] (uM)

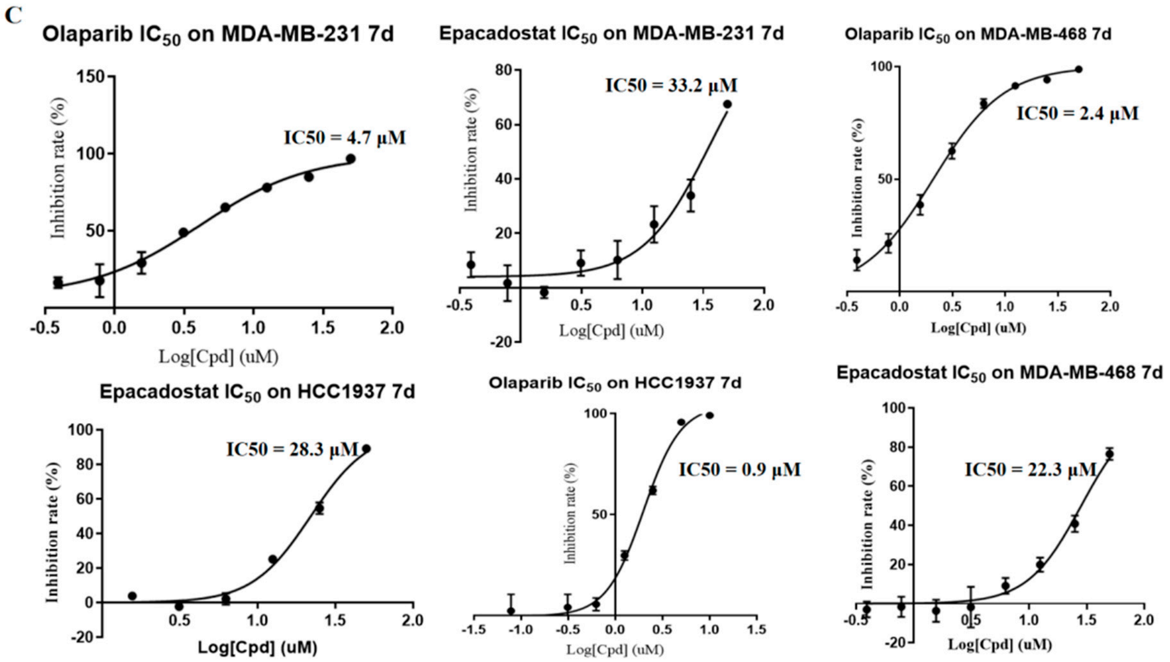

Figure S2. Drug-response curves of IC_{50} values for cell viability after a series of concentrations treatment of Olaparib or Epacadostat in the TNBC measured by CCK-8 assay. (A) The 48 h cellular proliferation curves of TNBC. (B) The 72 h cellular proliferation curves of TNBC. (C) The 7d cellular proliferation curves of TNBC.

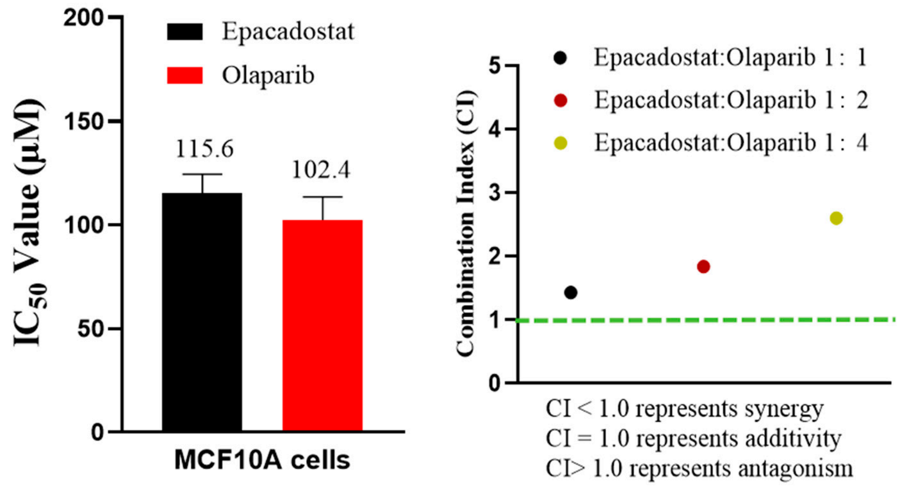

Figure S3. The synergistic effects of Epacadostat and Olaparib on the proliferation of MCF10A cells.

Table S1. CI values for the effects of combining Epacadostat with Olaparib on the proliferation of SW1990 cells after 7 days

Cell	SW1990	SW1990	SW1990

			Ratio (1:1)		Ratio (1:2)		Ratio (1:4)	
Group	Epacadostat (μM)	Olaparib (μM)	Epacadostat (μM)	Olaparib (μM)	Epacadostat (μM)	Olaparib (μM)	Epacadostat (μM)	Olaparib (μM)
Mean±SD	62.35±3.2	12.17±0.3	6.20±0.55**	6.20±0.25#	4.25±0.26**	8.50±0.06##	1.62±0.02**	6.28±0.26#
CI value	/	/	0.61±0.22		0.74±0.06		0.54±0.38	

**P<0.01 compared to Epacadostat group, #P<0.05 and ##P<0.01 compared to Olaparib group

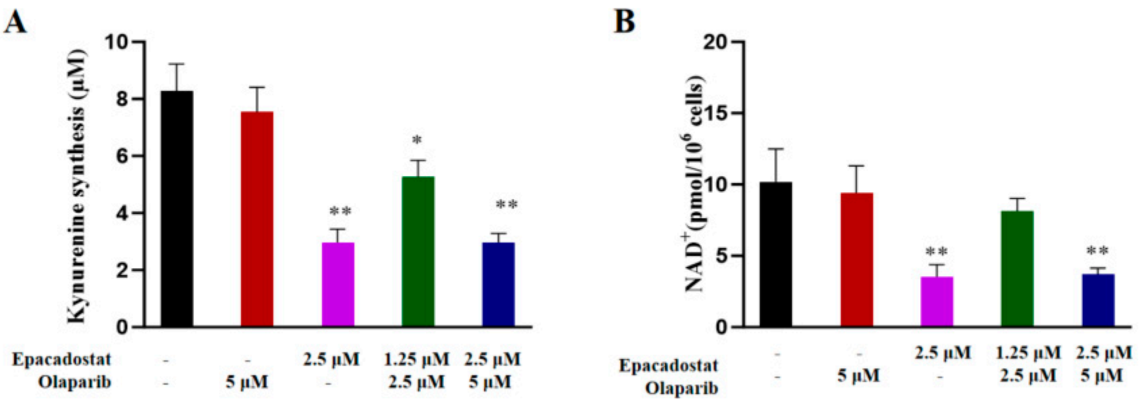

Figure S4. Epacadostat efficiently suppressed kynurenine and NAD^+ in MDA-MB-468 cells. (A) Concentrations of kynurenine were detected by colorimetry. (B) Concentrations of NAD^+ were detected by WST-8. All data were presented as means ± SD (n=3). Statistical significance of differences was determined by one-way ANOVA. **p < 0.01, *p < 0.05 compared with control group.

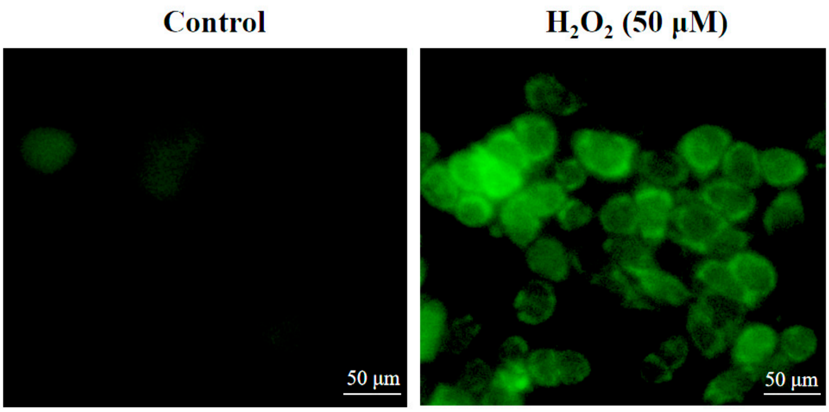

Figure S5. H_2O_2 induce intracellular reactive oxygen species (ROS) generation. ROS production was assessed by detection of DCFH fluorescence (green). Cells were also observed with phase-contrast micros-copy. Scale bar, 50 μm.

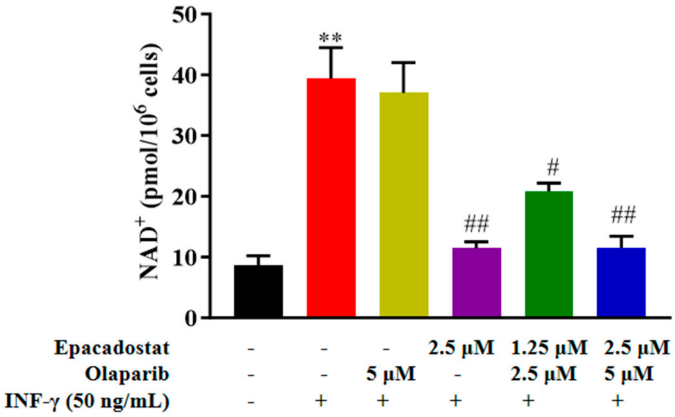

Figure S6. Epacadostat efficiently suppressed NAD^+ in MDA-MB-468 cells. All cells were pre-treated with IFN-γ (50 ng/mL) for 24 h. Subsequently, they were treated with Olaparib and/or Epacadostat for 2 days. All data were presented as means ± SD (n=3). Statistical significance of differences was determined by one-way ANOVA. $^{**}p < 0.01$ compared with control group, $^{\#\#}p < 0.01$ and $^{\#}p < 0.05$ compared with the IFN-γ (50 ng/mL) group.

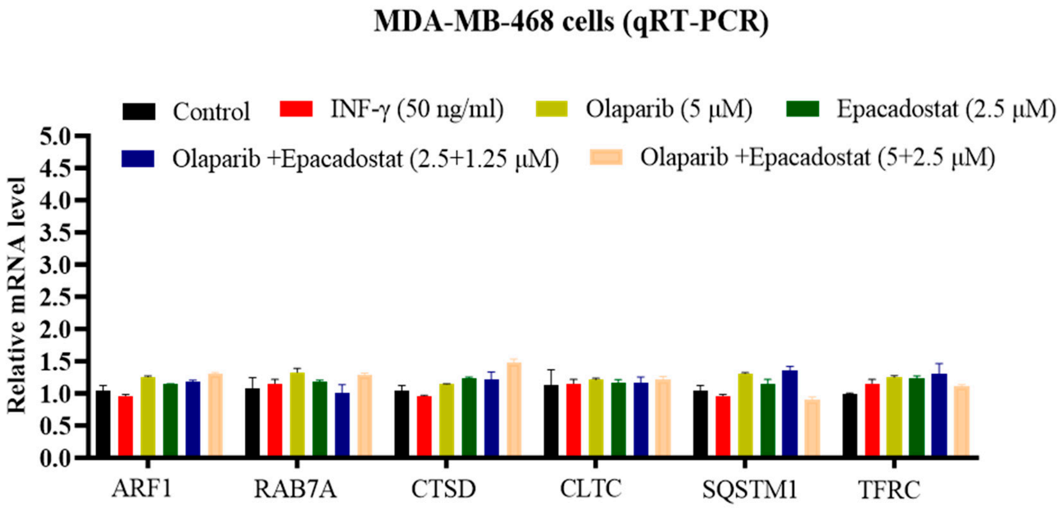

Figure S7. Quantitative PCR analysis of ARF1, RAB7A, CTSD, CLTC, SQSTM1, and TFRC mRNA levels following treatment with Epacadostat and/or Olaparib. Data were normalized to TBP and expressed as fold change relative to the control group.

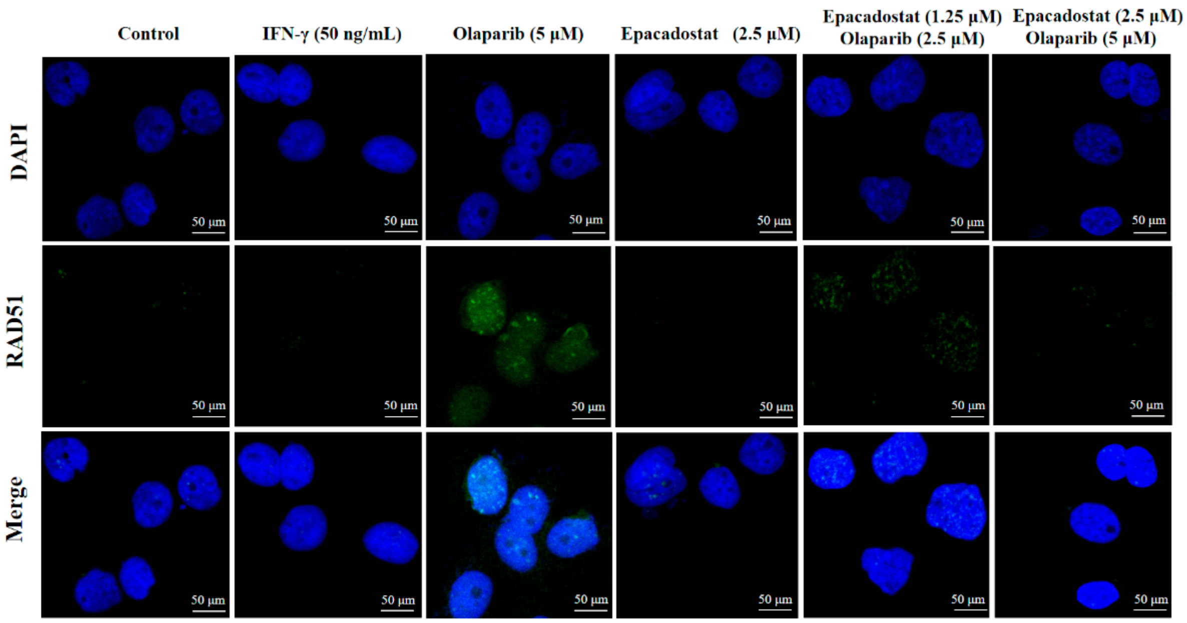

Figure S8. The effects of Epacadostat and/or Olaparib on RAD51 foci formation. Representative image of immunofluorescence staining of RAD51 foci 7 d after treatment with Epacadostat and/or Olaparib.
